# Evolutionary Analysis of Sex-Biased Gene Suggests Functional Conservation of Lifespan-Related Genes in Insecta

**DOI:** 10.3390/biology14091181

**Published:** 2025-09-02

**Authors:** Ziqi Cheng, Yueqi Lu, Hang Zhou, Yang Mei, Xi Chen

**Affiliations:** 1 College of Plant Protection, Jilin Agricultural University, Changchun 130118, China; chengziqi1004@gmail.com; 2 Yangtze Delta Region Institute (Quzhou), University of Electronic Science and Technology of China, Quzhou 324000, China; yueqi_lu@csj.uestc.edu.cn; 3 Key Laboratory of Biology of Crop Pathogens and Insects of Zhejiang Province, Institute of Insect Sciences, Zhejiang University, Hangzhou 310058, China; zhouhang716@zju.edu.cn; 4 Department of Clinical Laboratory, The Quzhou Affiliated Hospital of Wenzhou Medical University (Quzhou People’s Hospital), Quzhou 324000, China

**Keywords:** insects, sex-biased gene expression, phylogenetic analysis, *Tribolium castaneum*

## Abstract

Sex-biased gene expression is a fundamental driver of sexual dimorphism and plays an important role in evolutionary divergence across species. Here, we conducted a comparative evolutionary analysis of sex-biased genes (SBGs) in 13 insect species spanning four major orders: Hemiptera, Diptera, Lepidoptera, and Coleoptera. In total, 42,488 SBGs were identified, with low interspecific conservation—86.5% of orthogroups were shared by six or fewer species. Phylogenetic analyses showed that SBGs generally follow evolutionary trajectories similar to other protein-coding genes but show branch length differences suggestive of accelerated evolution in *Drosophila virilis* and *Tribolium castaneum*. Male-biased genes were more conserved than female-biased genes, which often showed reversed expression patterns, particularly in *T. castaneum*. Functional annotation of three rapidly evolving orthogroups in *T. castaneum* indicates possible associations with sex-specific traits such as lifespan, muscle development, and immunity, which should be considered hypotheses for future functional validation. These findings highlight the complex evolutionary dynamics of sex-biased genes and their contributions to phenotypic diversity and adaptive evolution in insects, providing a basis for future studies on the molecular mechanisms of sexual dimorphism and lineage-specific adaptations.

## Simple Summary

Males and females differ in traits such as morphology and lifespan, partly due to differences in gene expression between the sexes. We analyzed sex-biased genes across 13 insect species to examine their evolutionary patterns. Most of these genes were restricted to a few species, indicating rapid evolutionary turnover. Male-biased genes tended to be more conserved, whereas female-biased genes frequently showed reversals in expression. In *Tribolium castaneum*, we identified genes related to lifespan, muscle development, and immunity that had shifted in their expression patterns. These results provide new insights into the evolution of sex-biased gene expression and its contribution to insect adaptation.

## 1. Introduction

Sex-biased gene expression, which refers to differential expression between males and females, is a fundamental mechanism contributing to sexual dimorphism and shaping species’ evolutionary trajectories [1,2,3,4]. This sex-specific expression is shaped by multiple factors, including sexual selection, reproductive strategies, and ecological pressures, leading to evolutionary patterns distinct from those of unbiased genes [5,6]. Due to their species richness and ecological versatility, insects represent an ideal model for investigating the evolutionary dynamics of sex-biased gene expression [7,8,9,10,11,12,13,14,15].

Previous studies have shown that sex-biased genes often undergo rapid evolution, largely driven by sexual selection and sex-specific ecological pressures [16,17,18,19,20]. However, the extent to which these genes are conserved or divergent across species and evolutionary lineages remains poorly understood, especially in large-scale comparative studies across insect orders. A broad-scale analysis of sex-biased gene evolution in insects may reveal not only general principles of gene evolution but also lineage-specific ecological and evolutionary forces shaping sex-specific expression patterns [21,22,23,24].

To address these knowledge gaps, we analyzed sex-biased gene expression in 13 insect species representing four major orders: Hemiptera, Diptera, Lepidoptera, and Coleoptera. Our objectives were to (1) identify and characterize sex-biased genes in these species; (2) investigate the evolutionary conservation and divergence of sex-biased gene expression across species; and (3) assess the functional relevance of sex-biased genes exhibiting accelerated evolution in specific lineages.

A particular focus was placed on *Tribolium castaneum*, in which we observed a unique combination of reversed sex-biased expression and accelerated evolutionary rates. By exploring these patterns, we aim to elucidate how sex-biased gene expression contributes to trait diversification and adaptive evolution. This work provides a foundation for future studies into the molecular mechanisms underlying sexual dimorphism and ecological adaptation in insects.

## 2. Materials and Methods

### 2.1. Data Acquisition

All sex-biased genes were obtained from 13 insect species representing four orders—Hemiptera (*Acyrthosiphon pisum*), Coleoptera (*Tribolium castaneum*), Lepidoptera (*Bombyx mori*, *Danaus plexippus*), and Diptera (*Aedes aegypti*, *Anopheles albimanus*, *Anopheles gambiae*, *Anopheles stephensi*, *Drosophila melanogaster*, *D. pseudoobscura*, *D. simulans*, *D. virilis*, and *D. yakuba*)—based on the InSexBase database [25]. Genome annotation files and sex-biased gene expression profiles were retrieved from InSexBase and InsectBase 2.0 for subsequent analyses [25,26].

### 2.2. Orthogroup Clustering and Sequence Alignment

Orthologous relationships among sex-biased genes (SBGs) were inferred using OrthoFinder v2.5.5 [27]. Protein sequences of SBG orthologs were aligned using the MUFASA.py script from Seqrutinator v1.0 [28], incorporating MAFFT v7.525 [29] and HMMER v3.1 [30]. Subsequently, sequences with potential sequencing errors, gene model inconsistencies, or pseudogene origins were filtered out using the seqrutinator.py script. Both OrthoFinder and Seqrutinator were run with their default parameters (including an MCL inflation value of 1.5 in OrthoFinder), to ensure reproducibility. Biological replicates were already incorporated during the construction of the InSexBase/InsectBase expression datasets, and our analyses were based on the processed expression matrices provided by these databases. Therefore, no additional batch correction was required [25,26].

### 2.3. Phylogenetic Tree Construction and Visualization

Maximum likelihood phylogenetic analysis was conducted using IQ-TREE v2.2.2.9 [31], with the best-fitting amino acid substitution models selected by ModelFinder (integrated in IQ-TREE v2.2.2.9) [32]. Nodal support was estimated using the Shimodaira–Hasegawa approximate likelihood ratio test (SH-aLRT) test and ultrafast bootstrapping with 1000 replicates. The resulting phylogenetic trees were visualized using Interactive Tree of Life (iTOL) v6 [33] and FigTree v1.4.5 http://tree.bio.ed.ac.uk/software/figtree/ (accessed on 23 March 2025).

## 3. Results

### 3.1. Summary of Insect Sex-Biased Genes

**Figure 1:**
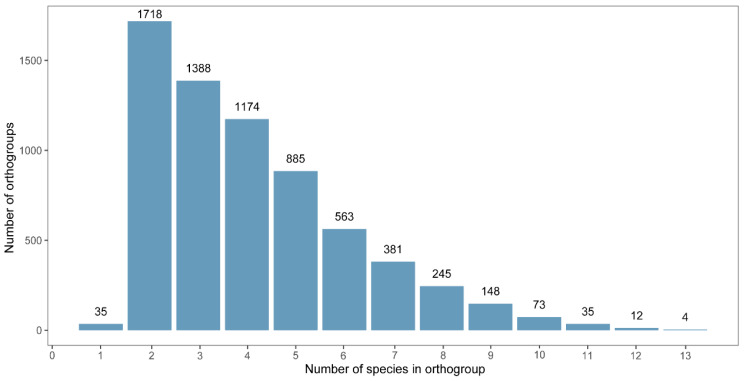
Summary of SBG orthogroups.

A total of 42,488 sex-biased genes (SBGs) were identified from 13 insect species. Homology-based clustering using OrthoFinder grouped these genes into 6661 orthogroups. Among them, 35 orthogroups were identified as species-specific, meaning that they contained genes from only one species (111 genes in total; Figure 1). In contrast, four orthogroups were shared by all 13 species, representing the most conserved set across our dataset. Additionally, 181 orthogroups contained single-copy genes that were consistently conserved across all four insect orders.

### 3.2. Phylogenetic Analysis

**Figure 2:**
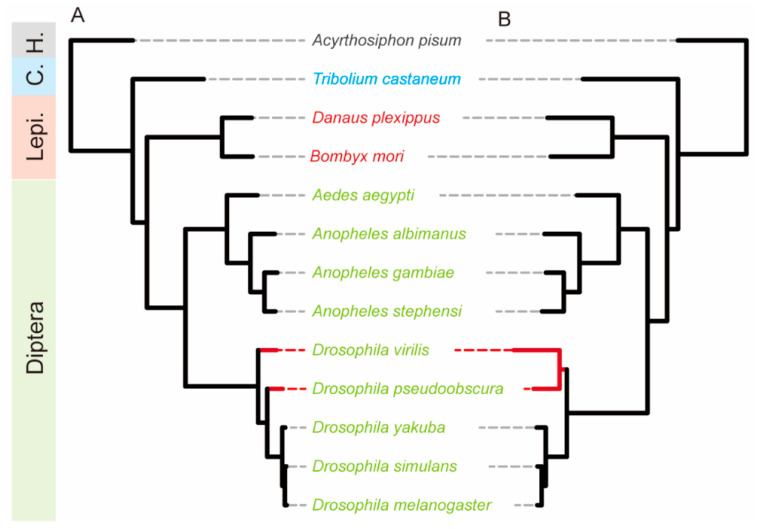
Phylogenetic comparison based on two gene sets. (**A**) Phylogenetic tree based on protein coding genes; (**B**) phylogenetic tree based on SBGs. Species name colors represent different insect orders (left labels defined as: H., Hemiptera; C., Coleoptera; Lepi., Lepidoptera; Diptera). Red branches highlight topological discrepancies between the two trees.

To compare the evolutionary dynamics of sex-biased genes (SBGs) with general protein-coding genes, two phylogenetic trees were constructed using single-copy orthologs. A total of 1524 SBG orthologs present in at least five species were used to construct a phylogeny of 13 insect species (Figure 2B), while 1402 orthologs shared across all species were selected from the entire protein-coding gene set to build a corresponding reference tree (Figure 2A). Comparative analysis of the two trees revealed a high degree of topological congruence, suggesting that SBGs largely follow evolutionary trajectories similar to those of general protein-coding genes (Figure 2).

However, a notable topological discrepancy was observed between *Drosophila virilis* and *D. pseudoobscura*. In the protein-coding gene tree, these species formed a paraphyletic group, whereas the SBG-based tree supported a monophyletic relationship. This indicates that while SBGs may have distinct functional roles, their broader phylogenetic patterns remain largely consistent with those of general genes.

In contrast, comparison of branch lengths revealed substantial differences in evolutionary rates. In the SBG-derived tree, branch length differences among closely related species were relatively small (Figure 2B). In contrast, *D. virilis* exhibited significantly longer branch lengths in the protein-coding gene tree (Figure 2A), suggesting that sex-biased genes in this species may have undergone accelerated evolution, potentially reflecting a lineage-specific adaptive trajectory.

### 3.3. Evolutional Pattens of Conserved Sex-Biased Genes

To investigate the evolutionary conservation of sex-biased gene (SBG) expression, 181 single-copy orthologous gene families were identified. Among them, 12 gene families exhibited consistently male-biased expression across all four insect orders and were defined as “consistent male-biased single-copy orthologous gene families”. Notably, no gene families showed consistent female-biased expression, suggesting that female-biased patterns may be less evolutionarily conserved than male-biased ones.

In contrast, 169 gene families exhibited reversed sex-biased expression across different insect orders, with orthologs showing male-biased expression in some lineages and female-biased expression in others. These were categorized into two groups: non-consistent male-biased and non-consistent female-biased gene families. Specifically, 27 gene families were classified as non-consistent male-biased (i.e., male-biased in three orders and female-biased in one), while 44 were defined as non-consistent female-biased (i.e., female-biased in three orders and male-biased in one). Interestingly, non-consistent female-biased gene families were 63% more frequent than their male-biased counterparts, implying greater variability in female-biased expression. However, this trend may also reflect sampling bias among the sequenced species.

Further analysis of the 44 non-consistent female-biased gene families revealed that *Tribolium castaneum* exhibited sex-biased expression reversals in 18 cases, accounting for 40.9% of the total (Table 1). This frequency was substantially higher than that observed in the second-ranked species, *Bombyx mori* and *Anopheles gambiae*. In many cases where orthologs in other species were female-biased, *T. castaneum* showed a male-biased expression pattern. This distinct reversal trend highlights the lineage-specific dynamics of sex-biased gene expression in *T. castaneum*, offering novel insights into its evolutionary divergence.

### 3.4. Analysis of Sex-Biased Expression Reversal Genes

To further explore the phenotypic implications of sex-biased expression reversal, 18 orthologous gene families with reversed expression patterns in *T. castaneum* were analyzed. These genes showed male-biased expression in *T. castaneum*, in contrast to female-biased expression in other insect species.

**Figure 3:**
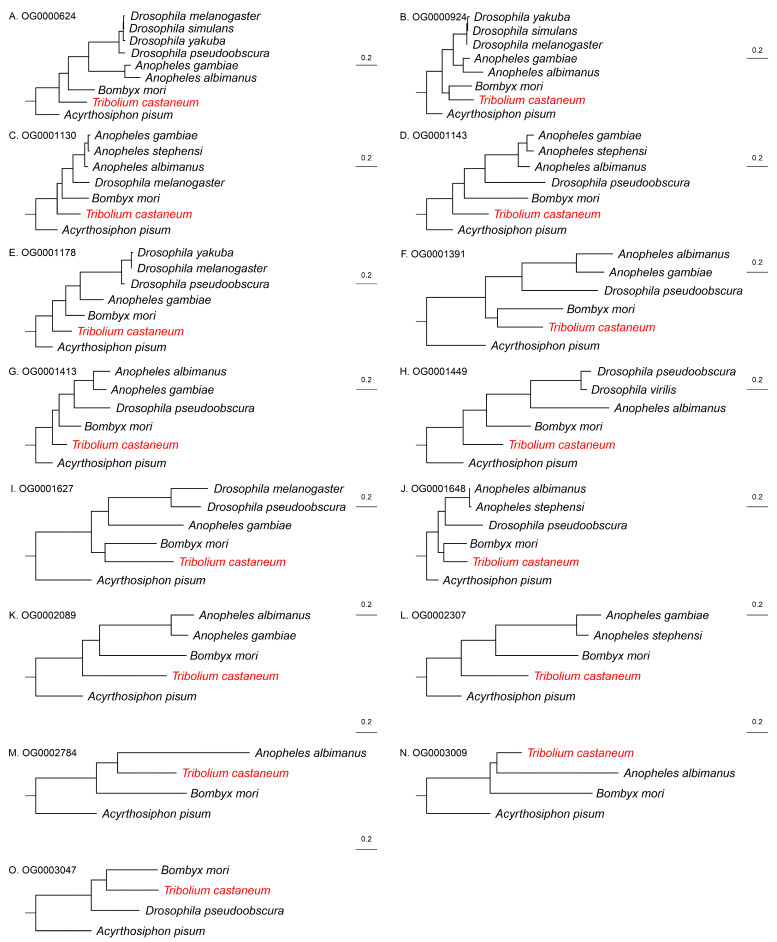
Fifteen non-consistent female-biased expression single-copy orthogroup gene trees, with the name of the orthogroup in the top left corner. Specific gene names are omitted in the trees, but species names are retained, and the species name of *T. castaneum* is highlighted in red. Scale bar indicates the number of substitutions per site (0.2).

**Figure 4:**
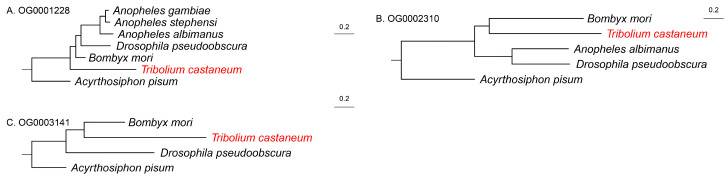
Three non-consistent female-biased expression single-copy orthogroup gene trees, with the name of the orthogroup in the top left corner. Specific gene names are omitted in the trees, but species names are retained, and the species name of *T. castaneum* is highlighted in red. Scale bar indicates the number of substitutions per site (0.2).

Phylogenetic trees were constructed based on these 18 gene families. In most cases, gene trees were congruent with the species-level phylogeny (Figure 3 and Figure 4), suggesting that the evolutionary trajectories of these genes largely mirrored species evolution. However, three gene trees exhibited distinct patterns (Figure 4). Specifically, gene trees for OG0001228, OG0002310, and OG0003141 showed substantially longer branch lengths for *T. castaneum* orthologs, indicating potential accelerated evolution in this species.

These findings provide new perspectives on the evolutionary significance of sex-biased expression reversal and offer insights into lineage-specific adaptations and evolutionary mechanisms contributing to biodiversity in *T. castaneum.*

### 3.5. Functional Analysis of Three Orthogroups with Accelerated Evolution in T. castaneum

A functional analysis was conducted for the three orthogroups exhibiting accelerated evolution in *T. castaneum*.

The first, OG0001228, corresponds to the proteasome activator complex subunit 3 (*PSME-3*) gene (*T. castaneum*, LOC663373) (Table 2). In both nematodes and humans, *PSME-3* has been shown to be closely associated with lifespan regulation [34]. High expression of this gene extends lifespan in nematodes and delays cellular aging in humans, whereas reduced expression shortens lifespan. Given the observed sex-biased expression of this gene in insects, we examined reported lifespan differences between sexes across various insect species. In taxa where *PSME-3* is female-biased (e.g., mosquitoes, fruit flies, pea aphids, and silkworms), females typically have longer lifespans. Conversely, in *T. castaneum*, where the gene is male-biased, males tend to live longer [35]. These observations are consistent with the hypothesis that *PSME-3* may play a conserved role in lifespan regulation and could contribute to sex-specific lifespan divergence in insects, a possibility that warrants further functional validation.

We analyzed the chromosomal locations of the OG0001228 orthogroup across various species and observed distinct patterns related to sex-specific expression and chromosome type. In species with XY sex determination, genes exhibiting female-biased expression are predominantly located on the X chromosome, as seen in *A. albimanus* (AALB006416), *A. gambiae* (*AgaP*_AGAP000308), *A. stephensi* (*Aste*1435_g), and *D. pseudoobscura (LOC4815668). In contrast, *T. castaneum* (LOC663373) shows male-biased expression, with its orthologous gene situated on an autosome, consistent with males having one fewer X chromosome than females. Similarly, orthologs in *A. pisum* (LOC100169004) and *B. mori* (KWMTBOMO04424) are also located on autosomes. These findings suggest that the evolutionary positioning of these genes may reflect a path of least resistance, adapting to the chromosomal architecture and sex-specific expression demands of each species.

The second orthogroup, OG0002310, corresponds to the non-SMC condensin I complex subunit G (*NCAPG*) gene (*T. castaneum*, LOC662437) (Table 3). In several avian species, *NCAPG* plays a key role in muscle development, influencing muscle mass and body size, and is a target gene in poultry breeding programs [36,37]. This gene may similarly contribute to sexually dimorphic traits in insects, particularly in body size and muscle development.

The third orthogroup, OG0003141, corresponds to the leucine-rich repeat containing 15 (*LRRC15*) gene (*T. castaneum*, LOC103314026) (Table 4). The human ortholog of *LRRC15* has been implicated in SARS-CoV-2 susceptibility, suggesting a potential role in antiviral defense. Sex-biased expression of *LRRC15* in insects could underlie differences in immune responses and disease resistance between males and females.

## 4. Discussion

Our findings provide new insights into the evolution and conservation of sex-biased gene (SBG) expression across four major insect orders: Hemiptera, Diptera, Lepidoptera, and Coleoptera. The identification of 42,488 SBGs and their classification into 6661 orthogroups revealed low evolutionary conservation, with 86.5% of orthogroups present in six or fewer species. This highlights the dynamic, lineage-specific nature of sex-biased gene expression in insects, consistent with previous studies demonstrating the rapid evolution frequently associated with such genes [38,39].

Phylogenetic analysis using 1524 single-copy SBG orthologs and 1402 single-copy orthologs from general protein-coding genes confirmed that SBGs largely follow evolutionary trajectories similar to those of typical protein-coding genes. The high degree of topological congruence between the two phylogenetic trees suggests that, while SBGs may have lineage- or function-specific roles, their overall evolutionary history is not distinct from other coding genes. However, discrepancies—such as the paraphyly of *D. virilis* and *D. pseudoobscura* in the protein-coding tree versus their monophyly in the SBG tree—suggest potential differences in selective pressures acting on sex-biased genes within specific lineages.

Additionally, an accelerated evolutionary rate of SBGs was observed in *D. virilis*, as evidenced by increased branch lengths. This may reflect adaptive responses to sex-specific selective pressures or ecological factors unique to the species. Similar patterns have been reported in other taxa, where sexual selection and reproductive conflict drive rapid evolution in sex-biased genes [40,41].

The absence of any single-copy orthogroups with consistent female-biased expression across all four orders—contrasting with twelve consistent male-biased families—supports the idea that male-biased gene expression tends to be more evolutionarily conserved. In contrast, the frequent reversals in female-biased expression, particularly in *T. castaneum*, may suggest relaxed selection or species-specific regulatory mechanisms affecting female-biased genes. These expression reversals may be linked to ecological or life-history traits that demand greater transcriptional plasticity in females.

In addition, dosage compensation mechanisms—by equalizing the expression of sex-linked genes between sexes—may also modulate the extent of observed sex-biased expression. Dosage compensation is well documented in *Drosophila* and has also been demonstrated in *A. stephensi* [42,43]. Differences among insect lineages in the presence or efficiency of dosage compensation may partly explain variation in the strength and chromosomal distribution of sex-biased genes. Moreover, sexual dimorphism in mosquitoes is reflected in feeding behavior, as only females are hematophagous and thus directly exposed to pathogens. This difference may shape immune responses and contribute to sex-biased gene expression in immunity-related pathways, as highlighted by previous studies [44]. Future studies that integrate these perspectives, together with expanded sampling and functional validation, will help clarify the mechanisms underlying sex-biased gene evolution.

## 5. Conclusions

In conclusion, this study demonstrates the complex evolutionary dynamics of sex-biased gene expression across four major insect orders. Although sex-biased genes generally follow evolutionary trajectories similar to other protein-coding genes, they exhibit lower conservation across species and show distinct lineage-specific adaptations. Branch length differences in specific sex-biased genes of *T. castaneum* may suggest lineage-specific ecological or evolutionary pressures, but require further molecular-evolutionary tests to confirm. Moreover, contrasting conservation patterns between male- and female-biased genes highlight divergent selective forces shaping sex-specific traits: male-biased genes appear more conserved, which may reflect stronger purifying selection linked to male reproductive functions, whereas the lower conservation of female-biased genes could be influenced by relaxed constraints or lineage-specific ecological factors.

These findings provide hypotheses regarding the molecular basis of sexual dimorphism and its possible role in shaping genetic and phenotypic diversity in insects. Future work should broaden taxonomic sampling and integrate functional validation to elucidate the regulatory and adaptive mechanisms underlying these patterns. Such efforts will provide deeper insights into how sex-biased gene expression drives evolutionary innovation and ecological diversification across insect lineages.

## Figures and Tables

**Table 1:** Turnover of female-biased expression.

Species	Number of Turnovers
*Tribolium castaneum*	18
*Bombyx mori*	7
*Anopheles gambiae*	7
*Drosophila pseudoobscura*	5
*Anopheles albimanus*	3
*Acyrthosiphon pisum*	3
*Anopheles stephensi*	1

**Table 2:** Annotation of OG0001228 orthogroup.

Species	Gene ID	Tissues	M-FPKM	F-FPKM
*A. pisum*	LOC100169004	whole body	51.345	168.215
*A. albimanus*	AALB006416	carcass	104.623	122.974
*A. gambiae*	AgaP_AGAP000308	carcass	63.344	193.023
*A. stephensi*	Aste1435_g	whole body	27.62	73.823
*B. mori*	KWMTBOMO04424	head	63.89	228.915
*D. pseudoobscura*	LOC4815668	whole body	7.62	29.735
*T. castaneum*	LOC663373	gonad	59.295	1.458

M-FPKM means FPKM in male samples, and F-FPKM means FPKM in female samples.

**Table 3:** Annotation of OG0002310 orthogroup.

Species	Gene ID	Tissues	M-FPKM	F-FPKM
*A. pisum*	LOC100169004	whole body	51.345	168.215
*A. albimanus*	AALB006416	carcass	104.623	122.974
*A. gambiae*	AgaP_AGAP000308	carcass	63.344	193.023
*A. stephensi*	Aste1435_g	whole body	27.62	73.823
*B. mori*	KWMTBOMO04424	head	63.89	228.915
*D. pseudoobscura*	LOC4815668	whole body	7.62	29.735
*T. castaneum*	LOC663373	gonad	59.295	1.458

M-FPKM means FPKM in male samples, and F-FPKM means FPKM in female samples.

**Table 4:** Annotation of OG0003141 orthogroup.

Species	Gene ID	Tissues	M-FPKM	F-FPKM
*A. pisum*	LOC103309830	whole body	1.72	9.645
*B. mori*	KWMTBOMO13951	gonad	7.865	8.465
*D. pseudoobscura*	LOC6901163	whole body	3.41	8.165
*T. castaneum*	LOC103314026	gonad	12.053	3.96

M-FPKM means FPKM in male samples, and F-FPKM means FPKM in female samples.

## Data Availability

Data will be provided as requested.
